# A randomized comparison of three data collection models for the measurement of parent experiences with diabetes outpatient care

**DOI:** 10.1186/s12874-018-0557-z

**Published:** 2018-09-20

**Authors:** Oyvind Bjertnaes, Hilde Hestad Iversen, Torild Skrivarhaug

**Affiliations:** 1 Norwegian Institute of Public Health, Nydalen, PO Box 4404, 0403 Oslo, Norway; 2 Oslo University Hospital, Oslo, Norway

## Abstract

**Background:**

The objective of this study was to compare three data collection methods for the measurement of parent experiences with hospital outpatient care for child and adolescent diabetes, based on a randomised national trial in Norway involving both pen-and-paper and electronic response options.

**Methods:**

The sample frame was patients registered in the Norwegian Childhood Diabetes Registry. Parents of patients were randomised into the following groups (*n* = 2606): group A, who were posted questionnaires with only a pen-and-paper response option (*n* = 859); group B, who were posted questionnaires with both an electronic and a pen-and-paper response option (*n* = 886); and group C, who were posted questionnaires with only an electronic response option (*n* = 861). The three groups were compared on response rate, background variables about respondents, main study results and survey costs. Statistical analysis included logistic regression to test group differences in response probabilities and multilevel linear regression to test differences in parent experiences.

**Results:**

The response rate was 61.8% for group A, 62.4% for group B and 41.6% for group C. The probability of answering was significantly higher for group A (OR = 2.3, *p* < 0.001) and B (OR = 2.3, *p* < 0.001) compared to group C. Respondent age, gender, education, living with the child and the degree of participation in consultations did not differ significantly between the three groups. Group differences in parent-reported experiences were small, varying from 1.0 (equipment and doctor contact) to 2.4 (outcome), on a scale from 0 to 100. Only one of 18 group differences was significant: the mixed group had significantly higher score than the electronic group on the organization scale (*p* < 0.05). The total cost of the electronic model was less than half the cost of the other models, and cost per response was 5.1 euros for the electronic model compared to 8.2 euros for group A and 7.6 euros for group B.

**Conclusions:**

The models with pen-and paper questionnaire included had more than 20% higher response rate than the model with an electronic-only response option. Background variables and parent-reported experiences were similar between the three groups, and the electronic model was the more cost-effective model.

**Electronic supplementary material:**

The online version of this article (10.1186/s12874-018-0557-z) contains supplementary material, which is available to authorized users.

## Background

Patient-reported experiences have become an important part of quality assurance in health care. The measurement of patient experiences is often conducted by means of a survey. One challenging task in surveys is to develop and establish data collection procedures, where a large set of practical, technical, financial, legal and methodological issues need to be considered. Post-discharge postal surveys are common in hospital patient experience surveys, but several other data collection methods are used, including telephone interviews, interactive voice responding and face-to-face interviews [1, 2]. Web-based surveys present the field with a new, potentially cost-effective and quick data collection method.

In national and other large-scale surveys of patient experiences, most available data collection methods are costly. However, previous research in this area has shown that the costs of including web-based surveys are low compared to postal pen-and-paper surveys [3–5], which is in line with findings from other research fields [6, 7]. Costs are a major challenge in large scale surveys such as in our context; one national survey of patient experiences with hospitals in Norway includes around 25,000 patients, and is highly resource demanding. Another potential advantage of including web-based surveys, at least compared to the traditional postal patient experience survey, is shorter data collection time [3]. In our experience, web-based surveys with access to e-mail addresses and embedded report procedures might produce survey results several months quicker than the traditional postal survey. Shorter time from measurement to presentation of results, combined with the potential for interim analysis in web-based surveys, is more sensitive to the need for updated results in quality improvement work. A systematic review of the literature found that timing was one of the barriers for use of patient experience data in quality improvement [8]. On the other hand, research has shown that web mode surveys have lower response rates than other modes, with examples of studies with response rates as low as 10–20% [3, 9, 10]. Lower response rates for web-based surveys compared to postal modes are in line with findings from the broader research community [11, 12]. Previous research has shown only marginal differences in patient experiences and satisfaction between patients in web-based surveys and other modes [3–5, 9, 10, 13], giving some support to the lack of association between low response rate for web-based surveys and the amount of non-response bias. However, low response rates threaten the legitimacy of surveys at the clinic and in the public domain, as well as the ability of the surveys to identify important differences in patient-reported experiences between providers and over time.

The standard data collection procedure in national patient experience surveys in Norway is post-discharge postal surveys, with pen-and-paper questionnaire included and an option to answer electronically. Only around 10% of the respondents have chosen to answer electronically in the national hospital survey [14], but this is a relatively old population and probably has lower Internet literacy than younger patient groups. In previous Norwegian patient experience surveys among patients visiting general practitioners and primary-care out-of-hour services, 18% [15] and 17% [16] of respondents, respectively, answered electronically. A previous randomised study among women who had recently given birth yielded 24% electronic respondents in the group who could choose between pen-and-paper and electronic response modes in both mailings [4]. Another group with only an electronic response option in the initial mailing yielded 68% electronic respondents, but at the expense of more than a 10% lower response rate [4]. The Norwegian studies document differences in web mode preferences between different patient populations, but they also show a rather modestly developed web mode preference overall in mixed method surveys. Despite these shortcomings, the results from the previous randomised experiment [4] and the potential gains from lower costs and shorter data collection time are important reasons for conducting more research into web-based surveys in national patient experience surveys in Norway.

In Norway, national quality registries are increasingly used for monitoring and improving the quality of healthcare services for specific patient populations. In recent years the government has included the measurement of patient-reported experiences and outcomes as criteria for judging the quality of registers. The Norwegian Childhood Diabetes Registry decided to establish a development and validation project on patient-reported experiences, in collaboration with the Knowledge Centre at the National Institute of Public Health. One of the primary goals of the project was to test different data collection models. The study was conducted among parents of children and adolescents with type 1 diabetes. Parents were randomised into the following data collection groups: i) postal with pen-and-paper questionnaire; ii) postal with pen-and-paper questionnaire and electronic response option; iii) postal with electronic response option. The objective of this study was to compare the three groups with regard to response rate, background variables for respondents, parent-reported experiences and survey costs. Based on previous research among patients we hypothesized that the electronic group would have poorer response rate, similar respondent characteristics and parent experiences, and better cost-effectiveness, than the two other data collection groups.

To our knowledge, this is the first randomized trial on data collection models in the user experience field with parents as the target group. Compared to most adult patient populations, parents of children are younger and probably have better digital skills. Thus, in spite of the strong general support for pen-and-paper preference in the patient experience literature, we expected a higher response rate for the electronic group and more equal mode preferences in our study than previous patient based studies.

## Methods

### Data collection

The sample frame consisted of patients registered in the national registry for child and adolescent diabetes. Eligibility criteria were patients 0–17 years of age registered with type 1 diabetes, and with a minimum of one outpatient consultation during the previous year. A total of 2606 patients were included, and parents of these patients were sent an invitation letter to participate in the study. The registry transferred data about the patients to the National Institute of Public Health, including their contact information as the basis for conducting the survey. Two reminders were sent to non-respondents. The first reminder was sent approximately 3 weeks after posting the initial request, the second approximately 3 weeks after the first reminder. Due to wrong addresses 80 patients were excluded.

### Intervention and randomization

The lack of e-mail addresses in the sampling frame precluded a comprehensive electronic data collection model, which is the same as most of the other studies in this field [4, 5, 9, 10]. Thus, the electronic group in the randomised experiment included postal with an electronic-only response option. The second randomised group was postal with both pen-and-paper and electronic response options. This mixed mode is the standard model in national patient experience surveys and has also been used previously in randomised trials in the patient experience field [4, 5]. The third randomised group was postal with a pen-and-paper questionnaire, which is one of the most commonly used data collection models in this field [1, 2].

**Fig. 1:**
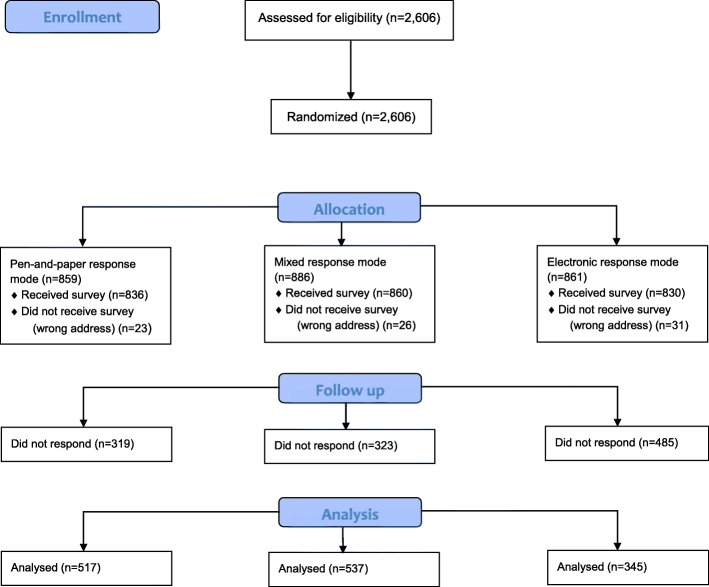
CONSORT Flow Diagram

Thus, parents of patients were randomised into the following groups: group A, who were posted questionnaires with only a pen-and-paper response option (*n* = 859); group B, who were posted questionnaires with both an electronic and a pen-and-paper response option (*n* = 886); and group C, who were posted questionnaires with only an electronic response option (*n* = 861). The different phases of the trial and flow for each randomized group are shown in Fig. 1.

### Questionnaire

We systematically searched and reviewed the literature, but did not identify a relevant and valid instrument to measure parent experiences with hospital outpatient care for child and adolescent diabetes. Thus, a development process was started to develop and validate a parent experience questionnaire. Qualitative interviews with 14 parents about health services at the outpatient clinics informed the first version of the questionnaire. The interviews had a particular focus on the most important health service issues from the parent perspective. Interview findings and a first version of the questionnaire were presented and discussed in a reference group for the development project, consisting of health personnel from the clinics, researchers and patient organizations. Based on the input from the reference group, the research group produced a revised questionnaire that was tested in cognitive interviews with 15 parents. The cognitive interviews showed that the questionnaire functioned well and only minor changes were made.

The questionnaire used in the randomized study consisted of four pages with 40 questions, and an additional page for comments relating to experiences with the outpatient clinic or to the questionnaire. Thirty-five questions related to experiences with services received from the outpatient clinic, while five were sociodemographic questions. Questions about parent-reported experiences were answered in a 5-point adjectival scale ranging from “not at all” to “to a very large extent”. The questionnaire is included in Additional file 1.

Exploratory factor analysis was used to assess the underlying dimensions of parent experiences. Principal axis factoring with oblique rotation (promax) was applied. Cronbach’s α coefficient was calculated to assess the internal consistency of the scales. The psychometric analysis resulted in the following six scales: organization (5 items), consultation (6 items), equipment (3 items), nurse contact (4 items), doctor contact (4 items) and outcome (5 items). All scales met the α-value criterion of 0.7, ranging from 0.71 (organization) to 0.90 (consultation). Items were linearly transformed to a scale from 0 to 100, where 100 represents the best possible experience. Experience scales were computed by averaging the items in a scale, and computation of scale scores for individuals required at least responses to half of the items in a scale.

### Cost estimates

The randomised study divided patients into three groups, but in future national surveys only one data collection procedure will be used for the whole sample. Thus, the cost calculations for each data collection model should be related to a full-sized national survey. Response patterns from the randomised experiment were used in these calculations, for instance, the pattern related to electronic responses before and after reminders. We estimated costs for printing and postage based on current market prices for the Norwegian Institute of Public Health, while estimates for resource use and salaries related to scanning questionnaires were based on actual use in previous surveys. Costs relating to infrastructure were not included, for instance, personal computers, scanner and electronic data collection system. The main outcome variables relating to costs were total estimated costs and cost per response for each data collection model.

### Statistical analysis

The difference in response rate between the three groups was tested with logistic regression analysis. We adjusted for patient’s age and gender, and conducted two regressions with varying reference categories to test all pairwise differences. Differences in respondent characteristics between the three groups were tested with Pearson chi-square tests for categorical variables and One-way ANOVA for continuous variables. Differences in parent-reported experiences between the three groups were tested with multilevel regression analysis. The multilevel model divides the total variance in parent-reported experiences into variance at the outpatient clinic (macro) level versus the individual (micro) level. The outpatient clinics were included as random intercepts, while data collection group, patient’s age and gender were included as fixed effects at the individual level. To test all pairwise differences we conducted six multilevel regressions with the electronic group as a reference category (one for each of the six parent-reported experience scales), then another six with the mixed group as reference category.

SPSS version 23.0 was used for statistical analyses, except cost calculations, where Microsoft Excel was used.

## Results

The response rate was 61.8% for group A, 62.4% for group B and 41.6% for group C (Table 1). The logistic regression analysis showed that the probability of answering was significantly higher for group A (OR = 2.3, *p* < 0.001) and B (OR = 2.3, *p* < 0.001) compared to group C. Most respondents in group B answered on paper, with 440 paper answers and 96 electronic answers. The initial response rate for the electronic group was around 10% lower than the other groups, and the remaining difference was primarily related to a lower effect of the second reminder.

**Table 1:** Respondents before and after each reminder for the three randomised groups, and final response rates

	Group A: Postal (*n* = 836)	Group B: Mixed (*n* = 860)	Group C: Electronic (*n* = 830)
Respondents before reminder			
Electronic, n	–	59	146
Paper, n	220	185	–
Response rate, %	26.3	28.4	17.6
Respondents after first reminder			
Electronic, n	–	21	118
Paper, n	140	109	–
Increase in response rate, %	16.7	15.1	14.2
Respondents after second reminder			
Electronic, n	–	16	81
Paper, n	157	146	–
Increase in response rate, %	18.8	18.8	9.8
Total			
Electronic, n	–	96 (17.9)	345
Paper, n	517	441 (82.1)	–
Response rate, %	61.8	62.4	41.6

The average respondent age was 43.0 for group A, 43.7 for group B and 43.3% for group C, none of the differences being significant (Table 2). Most respondents were female, 79.6% for group A, 77.8% for group B and 77.9% for group C (not significant). None of the other background variables differed significantly between the groups: education level, the percentage living with the other parent and the frequency of participation in consultations.

**Table 2:** Comparison of respondent characteristics for the three randomised groups

	Group A: Postal	Group B: Mixed	Group C: Electronic	Significance
Respondents’ age, mean (sd)	43.0 (6.8)	43.7 (6.1)	43.3 (6.2)	ns
Respondents’ gender, % women	79.6 (409)	77.8 (416)	77.9 (265)	ns
Respondents’ education, % (n)				ns
Elementary school	4.1 (21)	2.8 (15)	2.0 (7)	
High school	29.4 (150)	33.5 (179)	25.9 (89)	
University, 0–4 yrs	32.7 (167)	30.9 (165)	37.6 (129)	
University, > 4 yrs	33.9 (173)	32.8 (175)	34.4 (118)	
Respondent living together with other parent, % (n)	81.1 (413)	77.9 (415)	76.2 (256)	ns
Participation in consultations previous year, % (n)				ns
Never	2.0 (10)	0.9 (5)	0.3 (1)	
Once	4.3 (22)	4.5 (24)	4.4 (15)	
Twice	11.7 (60)	10.7 (57)	13.1 (45)	
3 times	27.0 (138)	29.8 (159)	29.4 (101)	
4 times or more	55.0 (281)	54.1 (289)	52.9 (182)	

^a^Pearson chi-square tests for categorical variables, One-way ANOVA for continuous variables

Group differences in parent-reported experiences were small, varying from 1.0 (equipment and doctor contact) to 2.4 (outcome), on a scale from 0 to 100 (Table 3). In the multilevel regressions, only one of 18 differences was significant: the mixed group had significantly higher score than the electronic group on the organization scale (df: 1377.8, t-value 2.1; p: 0.036.

**Table 3:** Comparison of parent-reported experiences between the three randomised groups

	Group A: Postal	Group B: Mixed	Group C: Electronic	Significance tests^b^		
				*A* vs *B*	*A* vs *C*	*B* vs *C*
Organisation^a^ (5 items)	73.3 (14.7)	74.0 (13.0)	72.3 (14.0)	ns	ns	*p* < 0.05
Consultation (6 items)	78.0 (18.3)	78.3 (16.9)	76.5 (18.7)	ns	ns	ns
Equipment (3 items)	65.5 (21.8)	65.2 (22.1)	64.5 (21.5)	ns	ns	ns
Nurse contact (4 items)	84.9 (14.5)	86.2 (13.0)	84.3 (13.6)	ns	ns	ns
Doctor contact (4 items)	84.2 (16.4)	84.7 (15.8)	83.7 (17.2)	ns	ns	ns
Outcome (5 items)	79.8 (18.0)	79.8 (17.3)	77.4 (20.2)	ns	ns	ns

^a^All scales are scored 0–100, where 100 is the best possible parent experience. Mean (SD)

^b^Multilevel regressions, adjusted for hospital level and patient’s age and gender

The total cost of the electronic model was less than half the cost of the other models, and costs per response was 5.1 euros for the electronic model compared to 8.2 euros for group A and 7.6 euros for group B (Table 4).

**Table 4:** Cost estimates for national survey based on data collection results in randomised study (*N* = 2600)

	Group A: Postal	Group B: Mixed	Group C: Electronic
Costs: (€)			
Printing	2676	2646	1397
Postage	9378	8868	4099
Salaries scanning	1050	870	0
Total costs	13,104	12,384	5497
Costs per response: (*€)*	8.2	7.6	5.1

## Discussion

The objective of this study was to compare three data collection methods, based on a randomised national trial in Norway involving both pen-and-paper and electronic response options. The study showed that the group with an electronic-only response option had more than a 20% lower response rate than the two other groups. However, the electronic group had similar respondent characteristics and parent-reported experiences to the two other groups, and the data collection model was the most cost effective.

Previous research on postal and web data collection in the patient experience field is scarce and heterogeneous, with variations in patient groups, interventions and outcome measures [3–5, 9, 10, 13]. The response rate for the web-only response option was below 20% for three studies [3, 9, 10], but increased substantially when combined with the pen-and-paper option, and over 60% in one study that mixed paper and web response options [5]. Our study had similar findings as previous research, with more than a 60% response rate for the postal and the mixed group, and a much lower response rate for the electronic group. As we expected, the response rate for the web-only response group in our study is much higher than web-only response rates in previous research [3, 9, 10], approximately 20–25% higher. The main reason for these differences is probably that our population was a rather young group of parents of children (average age around 43 years), compared to older and sicker patient populations in previous studies. The preference for pen-and-paper questionnaire compared to electronic questionnaire was slightly less in our study than previous studies, as indicated by the difference between the electronic group and the other groups. However, the 20% difference in response rate between the electronic group and the other groups in our study document the preference for pen-and-paper questionnaire also in younger populations.

In spite of large differences in response rates, the main results from previous studies show very minor differences in the level of patient experiences and satisfaction between web and postal respondents. Our study concurs with these findings: only one of 18 differences was significantly different when we compared the three groups. This is in line with similar research in this field [3–5, 9, 10], but in contrast to mode effects observed when comparing other data collection modes [17]. There might be several reasons for the lack of differences between pen-and-paper and electronic responses. First, the pen-and-paper and electronic questionnaire were designed to be as similar as possible, including, formulations, response categories, question order, lay-out and colours. The only difference relates to the ability to navigate back and forth in the questionnaire, including the possibility to have a full overview of the content of the questionnaire. A pen-and-paper questionnaire has full and immediate navigation possibilities, while the electronic questionnaire requires multiple mouse clicking and has limited possibility for questionnaire overview. However, these differences seem to have a small effect on how parents rate the clinics. Second, both the pen-and paper and electronic questionnaire were self-completed and sent to parents at their home, thus providing the same response context. Third, respondent characteristics for the three randomized groups were similar, including age, gender and education. All in all, the differences between answering electronically and on pen-and-paper were small. This implies that pen-and-paper and electronic questionnaires might be combined or replace each other in future surveys, without important effects on the measured level of parent experiences.

The purpose of national patient experience surveys in Norway is the systematic measurement of patient experiences as a basis for quality improvement, hospital management, free patient choice and accountability. The measurements are used as a basis for external quality indicators in a high-stake context of national quality indicators at an institutional level and in pay-for-performance schemes. This requires generalisability of findings and validity of measurement instruments, but also high performance of quality indicators based on the surveys. The latter includes face validity, adequate power at an institutional level, adequate case-mix adjustments and significant variation between institutions. The research and development project on parent experiences with hospital outpatient care for child and adolescent diabetes will assess these issues in future research, but the intended use of data in the future has direct consequences on the implications of the current study. Firstly, the standard sample size of 400 for institutions in national patient experience surveys in Norway is impossible to achieve with the number of patients and institutions in the Norwegian Childhood Diabetes Registry. With around 2600 patients and 27 institutions, the average number of patients per institution is lower than the national standard. Secondly, having lower institution samples than desired means every effort should be made to achieve a maximum response rate, including reviewing and using data collection procedures with documented effects on response rate [18]. Thirdly, even though a systematic review of the literature found positive attitudes to patient experience data among health personnel, a common engagement was not always present [19]. Methodological problems such as low response rates, and consequently large statistical uncertainty at an institutional level, might negatively affect the legitimacy of the survey, thereby undermining a common engagement and the use of data at the clinic. Taking into account the findings of the present study, and the future context of use of quality indicators, we do not recommend the electronic model. Instead, we recommend the mixed- data collection model; this model had the highest response rate of the three models, and was the most cost-effective of the two models with the paper questionnaire included.

The findings of this study has particular relevance for similar national or large scale surveys in Norway and internationally, but might not be valid outside this context, e.g. in clinical trials or local parent experience surveys. Furthermore, the technology available for electronic data collection affects the response rate. The response rate for the electronic group might have been substantially different with contacts through secure online systems or e-mail addresses, but this was not available in this study. For the three data collection models tested in this study, the response rate was not optimal, implying that implementing effective initiatives to increase the response rate is relevant for all models. A systematic review of trials on how to increase the response rate in postal and electronic surveys found a range of effective initiatives [18]: the most effective initiatives for postal surveys were monetary incentives, recorded delivery, a teaser on the envelope and a more interesting questionnaire topic. The latter was also one of the most effective initiatives in electronic surveys, in addition to non-monetary incentives, shorter e-questionnaires and including a statement that others had responded [18]. The available budget might be a constraint when it comes to the most expensive initiative(s), especially in large scale surveys, but several initiatives might be implemented without an increase in costs.

The main limitation of the current study was the lack of e-mail addresses in the sample frame. The implication was that even the electronic group had to include a postal invitation to the survey, adding to costs, and precluding the possibility of testing a comprehensive electronic data collection option. The same applies to previous studies, except for one study where e-mail addresses were available from electronic medical records [3]. This study ended up with a response rate of 14%, which indicates a substantial challenge in achieving an acceptable response rate in comprehensive electronic models. However, comprehensive web surveys are obviously the most promising model with regard to costs and survey time in future surveys in this area. Thus, new models of data collection will be developed and tested in future research, including models with access to and use of e-mail addresses. A second limitation of the current study is the lack of assessment of non-response bias [20], for instance, through follow-up studies of non-respondents [21, 22]. This is also an avenue for future research in this area.

## Conclusions

The models with pen-and paper questionnaire included had more than a 20% higher response rate than the model with an electronic-only response option. Even though background variables and parent-reported experiences were similar between the three groups, and the electronic model was the more cost-effective model, the low response rate for the electronic model poses a threat to the ability to provide adequate statistical power in settings with limited patient volume for each institution, and might negatively impact the legitimacy of the survey results at the clinic and in the public domain.

## Additional file

Additional file 1: Questionnaire. (PDF 258 kb)
