# Supplementary material for: A randomized comparison of three data collection models for the measurement of parent experiences with diabetes outpatient care
Source: BMC Med Res Methodol. 2018 Sep 20;18:95. doi: 10.1186/s12874-018-0557-z (PMC6149010; doi:10.1186/s12874-018-0557-z)
Supplement: Supplementary file 1 — Questionnaire. (PDF 258 kb) [file 12874_2018_557_MOESM1_ESM.pdf]

Norwegian Institute of Public Health

Your experiences with the children's outpatient clinic

The questions below concern your experiences with the children's outpatient clinic your child attends for diabetes.

Arrival and waiting

1. Are you and your child well received at the outpatient clinic?

- ☐ Not at all
- ☐ To a small extent
- ☐ To some extent
- ☐ To a large extent
- ☐ To a very large extent

2. Do you feel there's a lot of waiting at the outpatient clinic?

- ☐ Not at all
- ☐ To a small extent
- ☐ To some extent
- ☐ To a large extent
- ☐ To a very large extent

3. Do you find the waiting room satisfactory?

- ☐ Not at all
- ☐ To a small extent
- ☐ To some extent
- ☐ To a large extent
- ☐ To a very large extent

Organisation

4. Do you feel that the outpatient clinic is well organised?

- ☐ Not at all
- ☐ To a small extent
- ☐ To some extent
- ☐ To a large extent
- ☐ To a very large extent
- ☐ *Not applicable / Don't know*

5. Do you feel that the doctors and nurses cooperate well?

- ☐ Not at all
- ☐ To a small extent
- ☐ To some extent
- ☐ To a large extent
- ☐ To a very large extent
- ☐ *Not applicable / Don't know*

6. Do you feel that the person you have the appointment with is well prepared?

- ☐ Not at all
- ☐ To a small extent
- ☐ To some extent
- ☐ To a large extent
- ☐ To a very large extent
- ☐ *Not applicable / Don't know*

The nurses

7. Do you and your child see the same nurses every time you attend the outpatient clinic?

- ☐ Not at all
- ☐ To a small extent
- ☐ To some extent
- ☐ To a large extent
- ☐ To a very large extent
- ☐ *Not applicable / Don't know*

8. Do you and your child get enough time with the nurses?

- ☐ Not at all
- ☐ To a small extent
- ☐ To some extent
- ☐ To a large extent
- ☐ To a very large extent
- ☐ *Not applicable / Don't know*

9. **Do the nurses appear to know a lot about diabetes and diabetes treatment?**
- ☐ Not at all
- ☐ To a small extent
- ☐ To some extent
- ☐ To a large extent
- ☐ To a very large extent
- ☐ *Not applicable / Don't know*

10. **Do you feel that the nurses show care and concern for your child?**
- ☐ Not at all
- ☐ To a small extent
- ☐ To some extent
- ☐ To a large extent
- ☐ To a very large extent
- ☐ *Not applicable / Don't know*

The doctor

The questions below are about the doctor. If you see more than one doctor, please give us your overall assessment of all the doctors you see.

11. **Do you and your child see the same doctor every time you attend the outpatient clinic?**
- ☐ Not at all
- ☐ To a small extent
- ☐ To some extent
- ☐ To a large extent
- ☐ To a very large extent
- ☐ *Not applicable / Don't know*

12. **Do you and your child get enough time with the doctor?**
- ☐ Not at all
- ☐ To a small extent
- ☐ To some extent
- ☐ To a large extent
- ☐ To a very large extent
- ☐ *Not applicable / Don't know*

13. **Does the doctor appear to know a lot about diabetes and diabetes treatment?**
- ☐ Not at all
- ☐ To a small extent
- ☐ To some extent
- ☐ To a large extent
- ☐ To a very large extent
- ☐ *Not applicable / Don't know*

14. **Do you feel that the doctor shows care and concern for your child?**
- ☐ Not at all
- ☐ To a small extent
- ☐ To some extent
- ☐ To a large extent
- ☐ To a very large extent
- ☐ *Not applicable / Don't know*

More about what is discussed at appointments

15. **In your opinion, do the topics discussed at the appointments meet your child's needs?**
- ☐ Not at all
- ☐ To a small extent
- ☐ To some extent
- ☐ To a large extent
- ☐ To a very large extent
- ☐ *Not applicable / Don't know*

16. **Is it clear to you and your child what should be followed up before the next appointment?**
- ☐ Not at all
- ☐ To a small extent
- ☐ To some extent
- ☐ To a large extent
- ☐ To a very large extent
- ☐ *Not applicable / Don't know*

17. **Do you and your child have a say in what should be followed up before the next appointment?**
- ☐ Not at all
- ☐ To a small extent
- ☐ To some extent
- ☐ To a large extent
- ☐ To a very large extent
- ☐ *Not applicable / Don't know*

Being a parent/guardian at the clinic

18. **Are your views as a parent/guardian taken seriously?**
- ☐ Not at all
- ☐ To a small extent
- ☐ To some extent
- ☐ To a large extent
- ☐ To a very large extent
- ☐ *Not applicable / Don't know*

19. Do you get enough time for conversations without your child being present?

- ☐ Not at all
- ☐ To a small extent
- ☐ To some extent
- ☐ To a large extent
- ☐ To a very large extent
- ☐ *Not applicable / Don't know*

20. Are you given satisfactory information and guidance on how to follow up on your child's diabetes treatment?

- ☐ Not at all
- ☐ To a small extent
- ☐ To some extent
- ☐ To a large extent
- ☐ To a very large extent
- ☐ *Not applicable / Don't know*

21. Do you get the support you need to let your child take more responsibility for his or her diabetes treatment?

- ☐ Not at all
- ☐ To a small extent
- ☐ To some extent
- ☐ To a large extent
- ☐ To a very large extent
- ☐ *Not applicable / Don't know*

Information and training

22. Do you receive satisfactory information about the results of tests and examinations?

- ☐ Not at all
- ☐ To a small extent
- ☐ To some extent
- ☐ To a large extent
- ☐ To a very large extent

23. Do you receive satisfactory information about the development in your child's health and the risk of complications?

- ☐ Not at all
- ☐ To a small extent
- ☐ To some extent
- ☐ To a large extent
- ☐ To a very large extent
- ☐ *Not applicable / Don't know*

24. Do you receive satisfactory information from the outpatient clinic about available devices/equipment?

- ☐ Not at all
- ☐ To a small extent
- ☐ To some extent
- ☐ To a large extent
- ☐ To a very large extent
- ☐ *Not applicable / Don't know*

25. Do you and your child receive good training in managing the devices/equipment?

- ☐ Not at all
- ☐ To a small extent
- ☐ To some extent
- ☐ To a large extent
- ☐ To a very large extent
- ☐ *Not applicable / Don't know*

Availability

26. In your opinion, does your child have access to the best possible devices/equipment?

- ☐ Not at all
- ☐ To a small extent
- ☐ To some extent
- ☐ To a large extent
- ☐ To a very large extent
- ☐ *Not applicable / Don't know*

27. Does your child have satisfactory access to a nutritionist?

- ☐ Not at all
- ☐ To a small extent
- ☐ To some extent
- ☐ To a large extent
- ☐ To a very large extent
- ☐ *Not applicable / Don't know*

28. Does your child have satisfactory access to a psychologist?

- ☐ Not at all
- ☐ To a small extent
- ☐ To some extent
- ☐ To a large extent
- ☐ To a very large extent
- ☐ *Not applicable / Don't know*

29. Is it easy to get in touch with the outpatient clinic outside of appointments?

- ☐ Not at all
- ☐ To a small extent
- ☐ To some extent
- ☐ To a large extent
- ☐ To a very large extent
- ☐ *Not applicable / Don't know*

30. How do you feel about the number of appointments at the outpatient clinic?

- ☐ Too few
- ☐ A sufficient amount
- ☐ Too many
- ☐ *Not applicable / Don't know*

Usefulness

31. Do you feel that your child benefits from attending the outpatient clinic?

- ☐ Not at all
- ☐ To a small extent
- ☐ To some extent
- ☐ To a large extent
- ☐ To a very large extent
- ☐ *Not applicable / Don't know*

32. Do you, as a parent/guardian, benefit from attending the outpatient clinic?

- ☐ Not at all
- ☐ To a small extent
- ☐ To some extent
- ☐ To a large extent
- ☐ To a very large extent
- ☐ *Not applicable / Don't know*

Other questions

33. Does the follow-up at the outpatient clinic make you and your child more capable to live a good life with diabetes?

- ☐ Not at all
- ☐ To a small extent
- ☐ To some extent
- ☐ To a large extent
- ☐ To a very large extent
- ☐ *Not applicable / Don't know*

34. All in all, how dissatisfied or satisfied are you with how the outpatient clinic has followed up on your child and the diabetes treatment?

- ☐ Very dissatisfied
- ☐ Rather dissatisfied
- ☐ Both dissatisfied and satisfied
- ☐ Rather satisfied
- ☐ Very satisfied

35. All in all, how dissatisfied or satisfied are you with how the outpatient clinic has met you as a parent/guardian?

- ☐ Very dissatisfied
- ☐ Rather dissatisfied
- ☐ Both dissatisfied and satisfied
- ☐ Rather satisfied
- ☐ Very satisfied

Background questions

36. In the last year, how many times have you been present for all or part of your child's appointment?

- ☐ Never
- ☐ 1 time
- ☐ 2 times
- ☐ 3 times
- ☐ 4 or more times

37. Are you male or female?

- ☐ Male
- ☐ Female

38. What age are you?

☐☐ Number of years

39. What is your highest level of educational attainment?

- ☐ Primary school
- ☐ Secondary school
- ☐ Higher education/university (up to 4 years)
- ☐ Higher education/university (4+ years)

40. Do you live with the child's other parent/ guardian?

- ☐ Yes
- ☐ No
- ☐ *Not applicable*

Any additional comments about experiences with the outpatient clinic or comments on the questionnaire:

Thank you for taking the time to answer.

Norwegian Institute of Public Health
